# A Retrospective Study of Ethics Committee Monitoring Checklists of the Audiovisual Consent Process: An Ethical Perspective

**DOI:** 10.7759/cureus.34433

**Published:** 2023-01-31

**Authors:** Yashashri C Shetty, Raakhi Tripathi, Padmaja A Marathe, Sharmila Jalgaonkar, Snehalata Gajbhiye, Janhavi Katkar

**Affiliations:** 1 Pharmacology and Therapeutics, Seth GS (Gordhandas Sunderdas) Medical College & KEM (King Edward Memorial) Hospital, Mumbai, IND; 2 Institutional Ethics Committee, Seth GS (Gordhandas Sunderdas) Medical College & KEM (King Edward Memorial) Hospital, Mumbai, IND

**Keywords:** ethics committees, ethical review, consent process, regulations, clinical trial

## Abstract

Clinical trial regulations for new drugs in India released a gazette notification for obtaining audiovisual (AV) consent from all trial participants in November 2013. The reports of AV recordings of the studies from October 2013 to February 2017 submitted to the institutional ethics committee were analyzed in view of the Indian regulations on AV consenting. The reports of AV recording were checked: number of AV consents for each project, adequacy of AV recording, number of persons in the video, informed consent document elements (ICD) covered as per Schedule Y, confirmation of understanding by the participant, the time taken to complete the procedure, maintenance of confidentiality, and whether reconsent was taken. Seven studies of AV consent were monitored. Eighty-five (85) AV-consented and filled checklists were evaluated. The AV recording was not clear in 31/85, ICD elements were missing in 49/85 consents, time taken to complete the procedure was 20.03 ± 10.83 with the number of pages being 14.24 ± 7.52 (R= 0.29 p<0.041). In 19/85 consents, privacy was not maintained and on 22 occasions, reconsent were taken. There were deficits found in the AV consent process.

## Introduction

The year 2013 has been marked as the year that saw a series of regulations issued to streamline the ethical and scientiﬁc conduct of clinical trials in India. In the same context, on October 21, 2013, a directive from the Supreme Court of India was issued that mandated audiovisual (AV) recording of the informed consent process for all clinical trials. The National Regulatory Body for Indian Pharmaceuticals and Medical Devices, Central Drugs Standard Control Organization (CDSCO), together with the Drugs Technical Advisory Board (DTAB) then issued an order on November 19, 2013, stating that AV recording of the informed consent process (in addition to obtaining written informed consent) must be conducted for each study participant for all clinical trials [1,2]. The original order was subsequently modiﬁed, making AV consent legally binding only in cases of vulnerable populations involved in research on new chemical entities. A section was added in the same notiﬁcation that only audio consent (not AV consent) was needed in patients with HIV and leprosy. The purpose of the order on AV consent recording was to bring transparency to the process of consenting [3]. The AV recording of the informed consent was then made mandatory, including the preservation and archival of the AV recording while adhering to the principles of conﬁdentiality. This has been reinforced in the recent New Drug Clinical Trial Rules released on March 19, 2019 [4]. The process of the AV recording of informed consent is unique to India. However, the uniqueness does not invalidate its utility. The utility of this process can only be ascertained by critically examining it from the perspective of all stakeholders [5]. All the stakeholders (investigators, sponsors, regulators, and ethics committees) will face challenges while executing and monitoring the AV consenting process.

There is already a study published mentioning the challenges faced by stakeholders with AV consenting. There are many operational challenges like infrastructure issues, the ideal duration of the AV consent process, visibility of the physician and participant in the same video frame, breach of conﬁdentiality of participants, etc. [6]. Concerns were expressed by investigators and sponsors regarding the process of AV consenting, maintaining records, and their accessibility to stakeholders. In a study that recorded the perceptions of Indian investigators regarding the impact of new regulatory guidelines, 50% of them disagreed with the introduction of AV recording of informed consent [7]. On the contrary, another study from North India reported good experience with the AV consenting process in a vaccine trial [8]. A study from KEM Hospital, Mumbai, showed that AV recording of the informed consent process in a clinical trial appeared to improve the understanding of participants compared to those participants who were administered written informed consent alone [9]. Along with the directive for AV consent, there were expectations that investigator sites will formulate standard operating procedures (SOPs) to execute the AV consent process, keeping in mind the infrastructure requirements. A guidance document was also issued by CDSCO regarding the conduct of the AV consent process, which requires the Institutional Ethics Committee (IEC) to ensure and monitor that the investigators for regulatory studies meet the requirements [10]. Based on this guidance, the IEC of KEM created a checklist for monitoring AV consenting [11].

The IEC of KEM Hospital receives around 50 regulatory trials every year for review. As part of continued oversight, IECs conduct routine and ‘for-cause’ monitoring of the regulatory studies by visiting sites. With the new requirement of the AV recording of the consent process, IEC members had to review AV recordings too as a part of site monitoring. It was of interest to ﬁnd out how the investigators were implementing the new directive regarding AV consent recording. We could not ﬁnd any similar study wherein experiences of Ethics Committee (EC) members with respect to monitoring of the AV consent process were reported. It was perceived that sharing our experiences of AV consent process monitoring will add value and make the stakeholders aware of the challenges faced by the investigators. Hence, the present study was undertaken as a retrospective analysis of AV consent monitoring checklists available in the IEC oﬃce.

The objective of the study was to review and assess the AV consent monitoring checklists to capture practices (both accurate and inaccurate) followed by the Principal Investigator (PI) while implementing AV consent recording as required per the new regulatory norms.

## Materials and methods

The current study was a retrospective study, approved by the IEC (IECII/OUT/324/17) since it involved a document analysis of past IEC records with no identification of the names of the PIs, study title, department, patients, or any stakeholder involved. The studies for which monitoring was done to review whether AV consent was done as per the regulatory requirements were included. The AV consent monitoring checklists available in the IEC office, of the projects for which monitoring was conducted from October 2013 and February 2017, were analyzed. Strict confidentiality was maintained regarding the study protocols and the details of the investigators, sponsors, and IEC monitors while reviewing the data. The AV consent monitoring checklist prepared by the IEC members for this study was based on the CDSCO guidance document. The checklist of the IEC, which was used by the IEC members to monitor AV consent included the following points: 1. Number of AV consents monitored for each project; 2. Adequacy of AV recording: frame, video, and audio clarity; 3. The number of relevant persons (from the study team) in the video - their appropriateness; 4. Elements of the informed consent document (ICD) covered as per Schedule Y; 5. Review of confirmation of knowledge gained on the given trial by the participants; 6. Time taken to complete the procedure; 7. Issues regarding maintenance of confidentiality; and 8. An explanation of the need to re-consent due to an amendment in protocol/ICD. As per the IEC policy, AV consent monitoring findings with other site monitoring findings were communicated by the IEC to the respective PI for each study. A note of appreciation was also sent to the investigators showing good practices and a few recommendations were given by the IEC for a corrective action plan for AV consent in the future These have not been included in the present study. The data were entered in an adapted Microsoft Excel sheet (Microsoft Corporation, Redmond, WA) and GraphPad version InStat 3.10. Data were analyzed by descriptive statistics.

## Results

The IEC had monitored seven studies that had involved AV consent procedures as per the existing regulatory requirements and for which AV consent monitoring checklists were ﬁlled. All of these were Phase III pharma-sponsored studies. About 85 AV consents were monitored from these seven studies involving a total of 251 participants (25 to 50% participants from each study). Five studies were on the adult population (n=17), one on the pediatric population (n=48), and another study was conducted on pregnant women (n=20). About 22 reconsents were available and were reviewed from five studies.

The adequacy of AV recording was assessed as the recording video frame and audio clarity. For AV consenting, a dedicated room was visible in 54 of the 85 consents while in the other 31, the consent was taken in the oﬃce/ward/OPD. In the study where pediatric patients were recruited(n=48), the setup was not adequate in 46% of cases (22/48 consents). The child, as a participant, was not seen in the frame. The placement of the camera was not constant. It was ﬂexible and the angle changed frequently. In 15/85 (18%) cases, the frame was seen upside down. The video was clear in 81/85 and the storage of AV consent was adequate as per regulatory requirements in 71/85. However, the audio was not clear in 22/85 (26 %) cases.

The number of relevant persons from the study team present in the video and whether they were delegated and approved as per the IEC were also evaluated. There should be a formal introduction of the person conducting the informed consent discussion with the participant/legally authorized representative (LAR)/impartial witness involved in the informed consent process and he/she must provide information about the necessity for audiovisual recording. Table 1 depicts the lacunae as mentioned in the AV checklist prepared by the authors.

**Table 1:** Findings regarding clinical team information and choice of language

Sr no	Information missing	Number of deficiencies stated in the checklists
1.	Name and designation of the investigator	5
2.	Role of the investigator in the clinical trial	10
3.	Date and time not visible on the video	38
4.	Identity of the videographer who is part of the study team	37
5.	Language comfortable to participant	30

The AV recording permission was speciﬁcally sought in 49 cases and it was missing in 36 cases. There were 48 AV consents in the study involving the pediatric population; in 40 instances, the mothers posed as legally acceptable representatives who were accompanied by their mother-in-law, and when asked about the nominee, in 20 instances (out of 48 cases), women gave their name and the rest of the times, they gave their husband’s name. But in eight instances during AV consent, both parents were seen in the frame, the father was the LAR as well as the nominee. The study information was given to the patient in an inappropriate manner in 36 cases and some elements were missing in 49 instances out of the total 85 consents evaluated. The findings regarding the AV consent process are depicted in Table 2.

**Table 2:** Findings regarding lapses in the consent process in AV recording AV: audiovisual; IEC: institutional ethics committee

Sr no	Information Missing	Number of deficiencies stated in the Checklists
	Explanation regarding the study	22
	Consenting language mismatch	13
	Information on confidentiality and privacy	30
	Information on data sharing	43
	Risks /compensation to be paid for a study-related injury	5
	Right to withdraw	3
	Contact details of the principal investigator	30
	Contact details of IEC	25
9.	Nominee name	20
10.	Mismatch with the actual information mentioned in ICD	6

The AV recording was individualized in 74/85 cases. In 50% of cases, the participant read the consent document, and ample time was given to think and consent. Doubts were answered satisfactorily in 50%, and in 75% the process of obtaining signatures was seen. But in 44% of cases, the patient's understanding was not checked. The time required for a signature ranged from 0.42 mins to 12.18 mins. The duration of consent was 20.03 ± 10.83 minutes (n=85). The duration of re-consent was also 5.99 ± 3.3 minutes (n=22). In 21 cases, more than one session was undertaken (range 2-7) sessions. There were issues regarding the maintenance of conﬁdentiality, as multiple people were seen in the room (neighbors, mother-in-law, and husband and wife with their children). In 22% of the AV consent frames, privacy was not maintained, especially in the study with vulnerable participants. In 22 cases, there was re-consenting, but non-maintenance of privacy (n=2), and the reason for re-consenting not being explained (n=1) was detected. In the rest of the cases (n=19), the reconsent was satisfactory as per the checklist.

## Discussion

This was a retrospective analysis of AV consent monitoring reports of an IEC working at a tertiary care hospital. The IEC monitored all the studies involving AV consent procedures. This highlighted the due diligence on the part of the IEC, which continued to monitor the conduct of research studies. A total of 85 monitoring checklists were analyzed by the study team pertaining to seven regulatory studies. Eighty-five were initial AV consent and 22 were re-consent for protocol amendments.

The earlier regulation was CDSCO vide F. No. GCT/20/SC/Clin./2013 DCG1 dated 19.11.2013, which gave the direction that in all clinical trials, in addition to the requirement of obtaining written informed consent, AV recording of the informed consent process of each trial subject, including the procedure of providing information to the subject and his/her understanding on such consent is required to be done while adhering to the principles of confidentiality. The guidance provided directions regarding conducting the AV consent recording. It mentioned that all the essential elements in the patient information sheet, in addition to the requirement of obtaining written informed consent, the principle of privacy and confidentiality, consenting subjects for audio-visual recording, along with the procedure and quality of the recording, and the storage and archival of AV recordings should be included.

As gazette notification GSR 611, dated July 31, 2015, the directive was modified to implement AV consent only for a new chemical entity being evaluated in vulnerable populations in clinical trials, and a section was added that only audio recording is required for trials in patients having HIV and leprosy.

There were lots of reservations when the gazette notiﬁcation regarding the AV consent requirement came into force. There were issues regarding its feasibility as perceived by principal Investigators and acceptability by participants. Nowhere in the world, is AV consent mandated except in the US, wherein video recording is required only when the participant is illiterate [12]. A few stakeholders did perceive that it is a boon, as it safeguards both the participants and the investigators as stated by Kulkarni et al. [13].

In studies done by Figer et al. [9] and Gupta et al. [8], it was reported that the AV recording process enhanced the quality of the informed consent process considerably. In the study done by Figer et al., a total of 38 respondents (21 in the AV consent group and 17 in the written consent group) participated, wherein the total mean score of the AV consent group was significantly higher compared to that of the written consent group. Between the groups, the score was significant in the domains of rights and confidentiality. The proportion of participants who gave entirely correct answers was statistically significant in the domain of purpose. The time elapsed between the original consent and this study showed a weak inverse correlation. Similarly, the Gupta et al. study reported that the AV process ensured transparency and the accountability of the investigator, helped build better rapport, and thus provided a higher rate of recruitment. However, AV consent may be with hurdles as reported by the Chauhan et al. study, wherein the authors had assumed that there would be a refusal to participate as evident in 39% of participants who refused AV consenting [14].

The maximum number of patients monitored was in a clinical trial conducted on children (n=48) and pregnant women (n=20). In the vaccine study, we found a male preponderance for stating the name as nominee and found that women were uncomfortable when interrupted by the husband while asking questions, reﬂecting deviation from the principle of autonomy. In one study involving women, the patient information sheet was read from start to end without anything being explained. In the end, the participant was asked for comprehension and signature. The women never questioned the investigator. There can be two possibilities in such a scenario; ﬁrst, that the woman understood everything, or second, she is too inhibited or overdrawn to ask questions.

Ideally, before the commencement of AV recording, the investigator needs to take consent for the same, and this should be recorded and seen in the video. However, 42% of the time, consent was not taken as found in the checklist. It is possible that the PI had taken permission before starting the recording. As per the regulatory requirement, a dedicated room has to be given for AV consenting, wherein a camera is permanently ﬁxed for recording. In this study, the authors noted that in 36% of cases, the room was not constant in the pediatric trial, which can take a toll on participant privacy, while in other studies, the setup was adequate. The setup was again not adequate in the pediatric study because they could not engage the elder child, which could have come in the way of consenting procedures. Multiple people (residents, staff, MBBS students, other patients, and relatives as stated in the checklists) were seen in the frame, which can impinge on the issue of conﬁdentiality. The child as a participant was not seen in the frame and as per the guidelines, everyone involved in the trial - participants, their LAR, and an impartial witness - should be seen in the frame. The study by Shetty et al. mentioned in their AV consenting experience that this may not always be possible with a sick or irritable child and that can preclude AV consenting; if we want to show the child in the frame, it can be for a moment and then the child can be on his own or handed over to relatives. In 22% of the AV consent frames, privacy was not maintained especially in a study with vulnerable participants. This may have happened because there was no dedicated place for consenting and every time the place changed [6]. As per AV recording guidelines, the AV recording should be conducted in a room conducive to recording, with disturbance-free audio and video, the consent process. During the videography process, care should also be taken not to include unrelated persons/patients at the hospital within the ﬁeld of recording. The PIs were requested to refer to the SOP 5 of IEC dated July 26, which was effective from August 1, 2017, and had a guidance document on how to do AV consent recording.

A good practice was that the video was clear and stored properly, so the regulators and EC could monitor it whenever they wished, and it was as per the regulation. Another good practice was the individualization of AV recording in 74 cases. But the audio was not clear, which again raised doubts regarding the credibility/quality of the recording. The video recording of informed consent may not serve the intended purpose if the quality of the recording fails to meet the minimum standard required for the purpose. There should be a formal introduction of each person (person conducting the informed consent discussion participant/legally acceptable representative/impartial witness) involved in the informed consent process and information about requirements for audiovisual recording ‐ by name, designation, and his/her role in the research, current date and time, identity of videographer must be provided.

Consideration for language comfort was missing in the study. The AV recording frame also did not have a date and time, which leaves scope for manipulation.

The patient information sheet is an important document that the patient should read, understand, and comprehend and then volunteer to participate in the study. Complete information should be given to the participants based on which he/she will comprehend and volunteer to participate in the study. There were many missing elements in the patient information sheet as reported in the checklists, which were not conveyed to the patient. The participant’s autonomy and decision-making capacities are based on the information provided. In 50% of the participants, they read the consent document and ample time was given to them to think and consent, doubts were answered satisfactorily, and in 75% of cases, the signature procedure was also seen. But in 44%, the patient's understanding was not checked, which is mandatory as per the guidelines. The time required for signature ranged from 0.42 seconds to 12.18 mins. In one study, it was seen that the investigator gave the ICD to the patient, and the patient read the ICD in 2 minutes then the signature was asked by the PI and the participant obliged. There was absolutely no conversation between the PI and the participant (n=4, adult study). This again brings the scope of creating site speciﬁc SOPs for AV consenting, training the team in the consent process, creating the site facility, and sensitizing the participant in AV consenting as emphasized in the earlier Shetty et al. study [6]. However, this study did reveal that the implementation of the AV consent process is fraught with challenges (testing the participant's understanding and inadequacy of training to conduct the AV consent process, cultural sensitivity, and language barriers ). All stakeholders (investigators, sponsors, regulators, and ethics committees) associated with clinical trials need to join hands to ensure that the process of AV recording of the consent process complies with ethical norms.

In 21 cases, more than one session was required (2-7), which means that adequate time was given to the participant to comprehend and participate in those studies. Grady et al. raised concerns regarding the feasibility of time, sessions, visibility of face, audio recording, and maintenance of privacy during the AV consent process, and we also found a few deviations in these aspects too [15]. For the storage and archival of AV recordings in compliance with the existing regulatory requirements, the investigator faced hurdles. The IEC had made the monitoring checklists, which were modiﬁed based on the monitoring experiences of the EC members.

Subsequently, it was included in the IEC SOP 5 version v5. In 22 cases, there was reconsenting, but privacy was not maintained in two cases and the reason for re-consenting was not explained. At least here, the investigator had understood the situations where re-consenting has to be done. Ganguly and Ghooi had concerns regarding the execution of AV consenting, but our study reports a good experience [16,17].

The study does have limitations, as it is a retrospective analysis of the AV consent monitoring reports of a public hospital ethics committee so it may not be generalizable to other settings. The data are relevant only for India, as the rule for AV consenting is India speciﬁc and for the studies that qualiﬁes for AV consenting and not for routine consenting. The personal bias of the investigators cannot be ruled out.

AV consenting came into existence for increasing reliability and transparency, improving the quality of conduct of the informed consent process, and increasing society’s faith in clinical research. IEC and investigators are meant to follow this rule and because of this study, we have recommendations speciﬁc to each stakeholder. For investigators - creating site-speciﬁc SOPs, training the study team to conduct the AV consent process, maintaining the site facility, and sensitizing participants to the AV consent process. For the IEC, the SOP for the AV consenting review process and regular monitoring of the studies that involve AV consenting along with appropriate actions to be taken against defaulters with investigator education. Recommendations for regulators could be preparing a checklist for the AV consenting process and providing it to investigators who can implement AV consent effectively as per the requirement.

## Conclusions

The investigators were following the new regulation regarding AV consent but there was scope for improvement in the execution of the consent process. The study found that the facility was not adequate for AV consenting, audio was not audible, privacy was not maintained, and there was no adequate communication between the participant and the investigator team. The learnings from the study are important to all research stakeholders to streamline practices regarding the AV consent process.
